# Oxidative Stress in ICU Patients: ROS as Mortality Long-Term Predictor

**DOI:** 10.3390/antiox10121912

**Published:** 2021-11-29

**Authors:** Juan Carlos Ayala, Adriana Grismaldo, Luis Gonzalo Sequeda-Castañeda, Andrés Felipe Aristizábal-Pachón, Ludis Morales

**Affiliations:** 1 Faculty of Sciences, Pontificia Universidad Javeriana, Bogotá 110231, Colombia; 2 Department of Nutrition and Biochemistry, Faculty of Sciences, Pontificia Universidad Javeriana, Bogotá 110231, Colombia; mgrismaldo@javeriana.edu.co (A.G.); andres_aristizabal@javeriana.edu.co (A.F.A.-P.); 3 Department of Chemistry, Faculty of Sciences, Pontificia Universidad Javeriana, Bogotá 110231, Colombia; lsequeda@javeriana.edu.co

**Keywords:** reactive oxygen species, critically ill, sepsis, survival

## Abstract

Lipid peroxidation, protein oxidation, and mutations in mitochondrial DNA generate reactive oxygen species (ROS) that are involved in cell death and inflammatory response syndrome. ROS can also act as a signal in the intracellular pathways involved in normal cell growth and homeostasis, as well as in response to metabolic adaptations, autophagy, immunity, differentiation and cell aging, the latter of which is an important characteristic in acute and chronic pathologies. Thus, the measurement of ROS levels of critically ill patients, upon admission, enables a prediction not only of the severity of the inflammatory response, but also of its subsequent potential outcome. The aim of this study was to measure the levels of mitochondrial ROS (superoxide anion) in the peripheral blood lymphocytes within 24 h of admission and correlate them with survival at one year after ICU and hospital discharge. We designed an observational prospective study in 51 critical care patients, in which clinical variables and ROS production were identified and correlated with mortality at 12 months post-ICU hospitalization. Oxidative stress levels, measured as DHE fluorescence, show a positive correlation with increased long-term mortality. In ICU patients the major determinant of survival is oxidative stress, which determines inflammation and outlines the cellular response to inflammatory stimuli.

## 1. Introduction

Sepsis is well known as the leading cause of mortality in intensive care units (ICU) [1]. Sepsis is defined as a life-threatening condition associated with generalized organic damage due to the dysregulated immune response of the patient [2,3]. The pathogenesis is still not fully understood, however, there are two fundamental conditions within the inflammatory response: the inability of the cell to consume oxygen and the excessive production of oxidants [4], the latter being the cornerstone of the pathogenesis of the sepsis condition.

ROS are a set of unstable molecules, produced by all cells, which include hydrogen peroxide (H_2_O_2_), hydroxyl radical (•OH), singlet oxygen (^1^O_2_) and superoxide $O_{2}^{-}$. These molecules are involved in deoxyribonucleotide formation, prostaglandin production, and oxidation, carboxylation, and hydroxylation reactions that are essential for cell function [5]. ROS also participate in the defense of the host against microbial infections, in the regulation of vascular tone and cell adhesion reactions, and act as sensors for oxygen concentration [6]; in inflammation, ROS production is enhanced to act not only as inflammatory mediators but, more importantly, as regulators of cell signaling [7], promoting cell proliferation and cell survival or cell death.

Oxidative stress is the imbalance between the effectiveness of antioxidant defense and the rate of ROS generation, which causes an excess of oxidants within cells to an extant where this exceeds the oxidation/reduction rate of thiols, among others [8]. Under normal conditions, mitochondria are the main source of ROS in cells; in complex IV of the electron transfer chain, approximately 1 to 4% of the reactions occur in the presence of a defective reduction in oxygen to H_2_O, which results in the generation of $O_{2}^{-}$, as the most generated radical [9]. Phagocytes are another important ROS production system, given the presence of oxidant generators such as membrane-bound NADPH oxidase, which produces $O_{2}^{-}$ and myeloperoxidase in macrophages that convert H_2_O_2_ and Cl into Cl^−^, •OH and OH [7].

Furthermore, $O_{2}^{-}$ reacts with nitric oxide (NO), producing peroxynitrite, which interacts with mitochondrial components, leading to a variety of biological responses from the modulation of respiration to apoptotic cell death. ROS plays a key role as a signaling molecule in the pathogenesis of inflammation, which is considered to be an immunoregulator [9].

Once inflammation is triggered by Danger Associated Molecular Patterns (DAMPS) or Pathogen Associated Molecular Patterns (PAMPS), the mitochondrial electron transfer chain is inhibited by nitric oxide, overexpressed by the inflammatory stimulus with lipopolysaccharides (LPS) and the activation of the nuclear factor kB (NF-kB) [10], thus, interrupting the production of ATP while, at the same time, increasing the production of $O_{2}^{-}$. Additionally, NO and $O_{2}^{-}$ interact favoring and perpetuating mitochondrial dysfunction [11]. This mechanism explains the inability of cells to use oxygen, despite adequate VO_2_ tension, thus “cytopathic hypoxia” leads to multi-organ failure.

Markers of ROS production and antioxidant activity have been linked to several critical illnesses. Diseases such as cardiovascular disorders, and diabetes mellitus that affect critical illness are also linked to ROS formation and redox imbalance [6,12]. In addition, results from evidence-based research have linked oxidative stress to many ICU syndromes and diseases, including cardiogenic shock, sepsis, acute respiratory distress syndrome (ARDS), diaphragm fatigue, and burns [4].

Critically ill patients may have increased ROS levels or decreased antioxidant defenses. Many biological indicators of oxidative damage are being investigated in clinical trials, and the results can help clinicians determine whether ROS damage is occurring [13,14]. Thus, the objective of this study is to measure the levels of mitochondrial ROS in peripheral blood lymphocytes within 24 h of admission to the ICU, and to correlate them with patient survival status at 12 months after ICU and hospital discharge.

## 2. Materials and Methods

### 2.1. Study Population

This prospective observational study was conducted at the San Ignacio University Hospital in Bogotá, Colombia. Fifty-one (51) patients were included who were admitted to the ICU and did not present clinical signs of shock during the first 24 h. The patients were divided into two cohorts: septic and non-septic patients. The following inclusion criteria were taken into account: patients older than 18 years with two or more of the following signs of Systemic Inflammatory Response Syndrome; temperature 36 °C to 38 °C, heart rate greater than 90 beats/min, respiratory rate greater than of 20 breaths/min, partial pressure of carbon dioxide in the blood (PaCO_2_) at 32 mmHg, white blood cell count above 12 × 10^9^ cells/L or below to 4 × 10^9^ cells/L or the presence of more than 10% immature neutrophils, infectious diagnosis or positive blood culture.

Patients under 18 years of age, or those with known or suspected pregnancy, a body mass index (BMI) greater than 25, a family history of primary mitochondrial disease, treatment with drugs that could affect mitochondrial function and patients with chronic diseases such as hypertension, diabetes and cancer were excluded.

All patients enrolled in the study were followed up until day 28 and the survivors until 12 months after discharge from the ICU. Survival and mortality were recorded. The control group consisted of 10 healthy volunteers, matched by BMI with the patients and with no known morbidities.

### 2.2. Ethical Statement

The study was approved by the Hospital Universitario San Ignacio (HUSI), Institutional Ethics Committee (007890, 1 November 2015) and informed consent was obtained from all patients or family members, as well as from control subjects, before enrolling in the study.

### 2.3. Data Collection

The medical records provided demographic data, comorbid conditions, source of infection, laboratory, and microbiology results, use of vasoactive infusions, mechanical ventilation, antimicrobial administration, time in ICU, and vital status. These data were organized using a standard form for analysis. Sequential organ failure assessment (SOFA) and acute physiology and chronic health assessment (APACHE II) scores were calculated. 

### 2.4. Clinical Variables and ROS Production

Clinical variables such as serum lactate (mmol/L) and peripheral blood C-reactive protein (mg/L) were measured within 24 h of admission. To assess ROS production, a 10 mL aliquot of blood was drawn from the central line catheters as soon as possible once consent was obtained and no later than 24 h after admission to the ICU. The ROS production analysis was carried out on fresh samples for immediate collection. ROS production was measured in isolated lymphocytes with Ficoll-Hypaque (Sigma-Aldrich, St. Louis, MO, USA) and they were labeled with anti-CD45 antibody (Agilent, Santa Clara, CA, USA). Subsequently, flow cytometry and dihydroethidium staining (DHE, Abcam, Cambridge, UK) were used to assess the production of ROS in the lymphocytes of each patient. Cells from both populations were stained with 2.5 µM DHE for 30 min, washed with PBS, and acquired on the Guava^®^-EasyCyte^®^ 6-2L capillary cytometer (Luminex Corporation, Austin, TX, USA). Analysis of the mean fluorescence intensity for DHE was reported.

### 2.5. Statistical Analysis

Statistical analysis was performed with the SPSS statistic for Windows, Version 25 (IBM Corp, Armonk, NY, USA). A student’s *t*-test was performed to assess whether there were significant differences in the physical variables between ICU patients and the distribution of inflammatory and mitochondrial function markers. The easyROC: a web-tool for ROC curve analysis (V. 1.3.1) performed the ROC analysis, and the cut-off method used was the Youden method. Logistic regression analysis was used to evaluate the univariate and multivariate association between the septic status (positive versus negative) and the markers of inflammatory and mitochondrial function. The odds ratio (OR) was used as an index of association strength and the 95% confidence limits (95% CL) were calculated. Statistics with *p* < 0.05 were considered statistically significant. All statistical tests were two-tailed.

## 3. Results

Fifty-one (51) patients were recruited from the ICU; 22 septic and 29 non-septic patients. All patients (septic and non-septic) presented with inflammatory or postoperative clinical conditions (abdominal, cardiac, or orthopedic surgery), acute respiratory failure and trauma. Furthermore, the septic patients had abdominal sepsis or pulmonary sepsis. A total of 18 (35.2%) patients died within 12 months of follow-up (8 non-septic and 8 septic patients), with a total mortality of 27.6% in non-septic patients and 36.4% in the septic group. The physical characteristics, such as gender, age, and BMI of the study population did not show significant differences between alive and dead patients. The summary of these results is presented in Table 1. 

### 3.1. Inflammatory Markers

Conventional inflammatory clinical markers were analyzed in septic and non-septic patients. C-reactive protein (CRP) showed a significant increase in septic patients compared to non-septic patients (17.07 ± 2.7 and 15.31 ± 3.3, respectively; *p* = 0.047). Additionally, mixed venous oxygen saturation (Sv0_2_) also showed a significant increase in septic patients compared to non-septic patients (76.05 ± 12.3 and 68.72 ± 11.3, respectively; *p* = 0.035). These results confirm their accuracy as predictors of the severity of inflammation (Table 2). Other inflammatory markers did not show differences when comparing septic and non-septic patients. Lactate was not a strong predictor of inflammation in this study, and neither were leucocytes. All patients had APACHE and SOFA scores higher than the severity cutoff values, confirming the critical condition of each patient, however we did not observe any difference in relation to the sepsis status. 

We explored the effect of CRP, leucocytes, lactate, Sv0_2_, SOFA and APACHE as potential mortality predictors. We did not find any significant correlation between these inflammatory markers and the survival status in the patient group (Table 2). In addition, we performed a differential analysis to identify the sepsis status effect in mortality and inflammatory markers distribution. We found similar results for CRP, leucocytes, lactate, Sv0_2_, SOFA and APACHE in alive and dead patients from both the septic and non-septic groups. All the data are presented in the Table 3.

### 3.2. ROS Production and Survival

**Figure 1:**
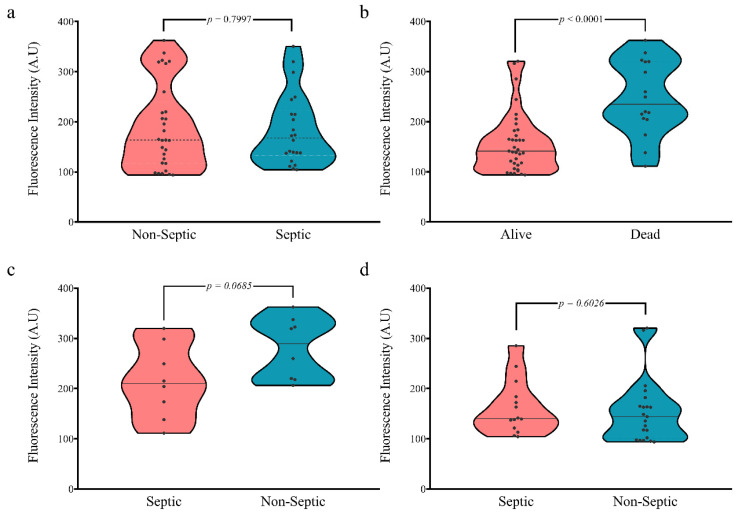
ROS production in the study group. (**a**) ROS production between septic and non-septic patients; (**b**) ROS production regard to survival status; (**c**) ROS production in dead patients; (**d**) ROS production in alive patients.

The ROS production was evaluated upon admission to ICU in each patient. First, we examined the ROS production related to sepsis status. Our results showed no significant difference between ROS production in septic and non-septic patients (mean of fluorescence intensity 183.66 and 189.39, respectively; *p* = 0.7997; Figure 1a). Interestingly, we found a significant increase in the ROS production in patients who died in contrast to those who survived (247.28 and 139.33, respectively; *p* < 0.0001; Figure 1b).

We performed an additional analysis to evaluate the sepsis status effect in the ROS production, with regard to survival. We found the sepsis status had no significant effect on mortality. Patients who died showed there was no significant increase in the ROS production of non-septic patients (280.8 and 213.8, respectively; *p* = 0.0685; Figure 1c). Similarly, patients who were alive showed similar ROS production values regardless of their sepsis status (Non-septic patients 154.6 and Septic patients 166.4; *p* = 0.6026; Figure 1d). These results present ROS production as a potential mortality marker.

**Figure 2:**
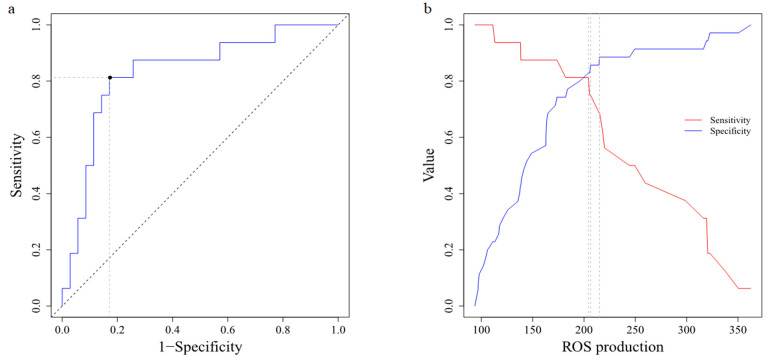
ROS biomarker features. (**a**) ROC curve with AUC 0.8321. (**b**) Sensitivity and specificity curve. The intercept denotes cut-off value for ROS production (204.22).

**Figure 3:**
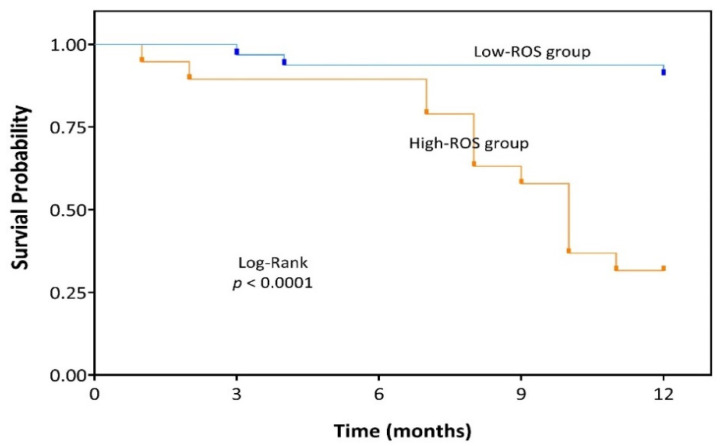
Kaplan-Meier analysis of UCI patients that had shown Low-ROS level (Blue line) and High-ROS level (Orange line); ROS, Reactive Oxygen Species.

The ROC curve showed that the optimal cut-off value of ROS production to distinguish the prognosis of UCI patients was 204.22 with AUC of 83.215% (Figure 2a,b). As a survival marker, the ROS production in the peripheral blood displayed a sensitivity of 81.2% (95% CI 54.4–96.0), specificity of 82.9% (95% CI 66.4–93.4). All other characteristics of ROS production as a biomarker are summarized in Table 4. According to the cut-off, UCI patients were divided into High-ROS and Low-ROS groups. A total of 32 UCI patients were Low-ROS and 19 UCI patients High-ROS. We found no difference between the groups regarding their physical or clinicopathological characteristics, however the survival status did show a significant difference (Table 5). Univariate Cox regression analysis proved that High-ROS production was a risk indicator for mortality in UCI patients (OR = 20.94; 95% CI 4.52–96.97; *p* < 0.0001). Kaplan-Meier analysis indicated that the High-ROS group had a worse overall survival (OS) (*p* < 0.0001; Figure 3).

## 4. Discussion

Long-term mortality in critically ill patients is an important issue, and one of the associated critical factors is the frailty of the patient [15]. The adult ICU population over 60 years of age is increasing, and this imposes a higher rate of morbidity and mortality [16] as seen in our cohort of patients with a median age of 64.1.

In the present study, it was found that the increase in ROS levels, mainly superoxide anion, in the first 24 h of admission to the ICU is a strong predictor of long-term mortality in non-obese elderly patients without morbidity, suggesting oxidative stress is the hallmark, not only of the inflammatory response, but also of the aging process.

There is a decline in cellular and organ function and reserve related to age, which leads to a reduction in the ability to respond to internal or external inflammatory stimuli favoring inadequate outcomes, and this condition is defined as frailty [17]. Thus, fragility is considered the consequence of the interaction between the aging process and some chronic diseases and conditions that prevail in the elderly [18]. Frail patients are characterized by a heterogeneous combination of reduced mobility, weakness, reduced muscle mass, poor nutritional status, and decreased cognitive function, and its prevalence is around 30% in patients above 60 years old. 

Oxidative stress is now recognized as the main feature in frailty, promoting inflammation in these patients despite diseases or any other state, revealed by the increased inflammatory parameters, particularly CRP and IL-6 [17]. In this sense, oxidative stress, frailty, and inflammation are associated with increased rates of morbidity, mortality, rates of hospitalization and long-term disability and long-term mortality [19].

ROS are now recognized as key physiological signaling molecules with regulatory functions. The physiological elevation of ROS generates responses that contribute to cellular homeostasis, while the unmodulated excess amount of ROS is responsible for the oxidative damage to the molecular and cellular structures. These processes have been somehow related not only to aging itself, but also to its clinical manifestations in the form of age-related diseases [20]. Interestingly, oxidative stress and inflammation appear to play an important role in these characteristics of aging [21].

Emerging evidence suggests that the high level of acute inflammation associated with critical illness does not completely resolve in some ICU survivors, and furthermore, that persistent inflammation may drive frailty-related disability and mortality in these patients and the cornerstone of the persistent inflammatory state could be the basal oxidative stress of the patient [22]. 

The oxidative injury contributes to the functional deterioration of different tissues and organs and, depending on the resilience of these systems, specific clinical alterations are manifested. For example, age-specific isolated depletion of functional reserve in the brain would lead to cognitive decline [23], whereas a dysfunctional kidney could lead to chronic kidney disease [24], a dysfunctional cardiovascular system to chronic vascular disease, or a dysfunctional lung to Chronic Pulmonary disease [25]. 

In ICU patients, the long-term consequences of oxidative injury include cognitive impairment and a loss of function of the cardiovascular and respiratory systems [26]. Therefore, critical illness predisposes older patients to the premature onset of frailty. 

Of course, fragility encompasses, then, an organic failure due to chronic or persistent oxidative stress and a chronic inflammatory condition, these being responsible for the signs of identity of age mentioned above (genomic instability, mitochondrial dysfunction, reduced proteostasis [27], alteration of the adaptive response in a cell in senescence, among others) which in turn decrease the resilience of the organs and increase the susceptibility to generalized failure.

In addition, aging is associated with alterations in redox signaling at the skeletal muscle level. Muscle contraction causes free radical generation. Fibers respond to contractile activity by enhanced superoxide and nitric oxide production leading to consecutive formation of ROS and nitrogen species [28]. 

There is an acceptance that the main source of ROS generated by muscle contraction is the mitochondria, but NADPH oxidase and xanthine oxidase are also important sources of ROS in skeletal muscle. ROS are key mediators in exercising muscle exerting an adaptive response that involves the transcription of redox-sensitive factors, which promote the increase in cytoprotective proteins such as catalase, superoxide dismutase and heat shock proteins that prevent oxidative damage. This response to ROS generation is severely attenuated in aged muscle damage [29]. 

The impaired ability of muscle cells to remove dysfunctional mitochondria can contribute to enhanced ROS production, which results in progressive mitochondrial dysfunction, thus creating a vicious cycle [29]. Oxidative injury contributes to faster age-induced decline in Type II fibers with lower mitochondrial content that are more susceptible to atrophy than the Type I fibers with a high mitochondrial content [30].

Another important issue of chronic oxidative stress after frailty is the effect on skeletal muscle, influencing not only its function but also its quality and muscle mass, and is a condition known as sarcopenia [31]. Increased levels of oxidative stress have been said to play a role in the muscle changes related to aging and sarcopenia. This phenomenon is also common in the post-ICU period, and muscle atrophy is associated with a poor prognosis [32].

Thus, the establishment of the presence and severity of oxidative stress in critically ill patients is crucial and can be determined through markers, generally measured in plasma and urine [33]. Among the biomarkers of oxidative stress can be found (1) the modifications induced by ROS in the proteins, which can cause damage to their structural integrity, induce loss of their activity, or even affect multiple metabolic pathways given their wide participation as regulatory molecules (2) increased expression of antioxidant defense systems and (3) functional changes in organelles. However, no studies have been found that measure the direct production of ROS, particularly superoxide anion in lymphocytes, in the acute onset of inflammation in critically ill patients, in their first 24 h in the ICU, with normal BMI and without chronic diseases, thus excluding bias factors due to a prior oxidative stress condition, as we did.

The data found that the patients who finally died in the following 12 months after hospital discharge were those with the highest levels of ROS in the first 24 h after the assault, with ROS levels being strong predictors of long-term mortality.

In this sense, and based on the data obtained in this study, long-term mortality undoubtedly depends on the amount of ROS production and the antioxidant or neutralizing capacity, showing the importance of ROS concentration in the prediction of the appearance of frailty in the post ICU period [34].

## 5. Conclusions

To our knowledge, this is the first study that not only measures ROS production in vivo in the first 24 h of admission to the ICU for septic and non-septic conditions, but also follows hospital discharge up to 12 months later, finding that an increase in ROS production in the first 24 h is a strong predictor of mortality. In other words, patients with low to moderate ROS production are more likely to survive after critical illness with hospitalization in the ICU.

## Figures and Tables

**Table 1:** Physical characteristics of the study group.

Characteristics		Non-Septic (*n* = 29)			Septic (*n* = 22)		
		Alive (*n* = 21)	Dead (*n* = 8)	*p*-Value	Alive (*n* = 14)	Dead (*n* = 8)	*p*-Value
Gender	Female	10 (47.6%)	7 (46.7%)	0.481 *	3 (21.4%)	4 (50.0%)	0.176 *
	Male	11 (52.4%)	3 (37.5%)		11 (78.6%)	4 (50.0%)	
Age (years)		59.24 ± 18.1	57.75 ± 24.8	0.860	57.14 ± 17.6	61 ± 20.2	0.644
BMI (kg/m^2^)		24.43 ± 2.9	23.46 ± 2.8	0.431	23.32 ± 3.3	23.05 ± 5.2	0.883

The values are shown as mean ± standard deviation; *p*-values were calculated by Student’s *t*-test; *, *p*-values were calculated by Fisher’s exact test. *p* < 0.05 was considered significant and the value is denoted in bold.

**Table 2:** Clinical inflammatory markers and ICU scores by mortality status.

Characteristics	ICU Patients (*n* = 51)					
	Non-Septic (*n* = 29)	Septic (*n* = 22)	*p*-Value	Alive (*n* = 35)	Dead (*n* = 16)	*p*-Value
CRP	15.3 ± 3.3	17.1 ± 2.7	**0.047**	16.1 ± 1.9	17.1 ± 4.1	0.112
Leukocytes	12.7 ± 7.2	13.4 ± 5.1	0.821	12.0 ± 2.1	15.2 ± 6.7	0.266
Lactate	1.9 ± 1.2	2 ± 1.1	0.642	2.06 ± 0.2	1.68 ± 0.2	0.280
SvO_2_	68.7 ± 11.3	76.1 ± 12.1	**0.035**	71.9 ± 13.0	71.7 ± 10.4	0.959
SOFA	8 ± 2.5	8.1 ± 4.1	0.991	8.4 ± 0.5	6.80 ± 0.7	0.093
APACHE	12 ± 5.6	14.4 ± 7.6	0.212	13.2 ± 1.1	11.93 ± 1.5	0.532

The values are shown as mean ± standard deviation; *p*-values were calculated by Student’s *t*-test; *p* < 0.05 was considered significant and the value is denoted in bold.

**Table 3:** Sepsis status effect in inflammatory marker distribution for mortality status.

Characteristics	Non-Septic (*n* = 29)			Septic (*n* = 22)		
	Alive (*n* = 21)	Dead (*n* = 8)	*p*-Value	Alive (*n* = 14)	Dead (*n* = 8)	*p*-Value
CRP	14.8 ± 1.7	17.8 ± 5.1	0.103	16.3 ± 2.1	18.5 ± 3.1	0.102
Leukocytes	11.6 ± 2.0	15.5 ± 6.8	0.445	12.6 ± 2.1	14.83 ± 7.9	0.478
Lactate	2.0 ± 0.3	1.55 ± 0.2	0.379	2.15 ± 0.3	1.82 ± 0.3	0.500
SvO_2_	67.5 ± 12.5	71.7 ± 6.7	0.382	78.5 ± 11.2	71.7 ± 13.7	0.225
SOFA	8.2 ± 0.6	7.38 ± 0.8	0.396	8.79 ± 1.1	6.14 ± 1.4	0.171
APACHE	11.9 ± 1.2	12.13 ± 2.1	0.926	15.14 ± 2.2	11.71 ± 2.3	0.339

CRP, C Reactive protein; SvO_2_, Mixed venous oxygen saturation; SOFA, Sequential Organ Failure Assessment; APACHE, Acute Physiology and Chronic Health Evaluation; the values are shown as mean ± standard deviation; *p* < 0.05 was considered significant and are denoted in bold; *p*-values were calculated by Student’s *t*-test.

**Table 4:** Characteristics of ROS as a biomarker in peripheral blood.

Statistics	Value	95% CI	
		Lower Limit	Upper Limit
Sensitivity	81.2	0.544	0.960
Specificity	82.9	0.664	0.934
Positive Predictive Value	68.4	0.469	0.922
Negative Predictive Value	90.6	0.727	0.966
Positive Likelihood Ratio	4.740	2.205	10.190
Negative Likelihood Ratio	0.226	0.081	0.635

CI, Confidence Interval.

**Table 5:** Physical and clinicopathological characteristic in low and high ROS groups.

Characteristics	Low ROS (*n* = 32)	High ROS (*n* = 19)	*p*-Value
Age (years)	60.39 ± 3.0	55.95 ± 5.1	0.428
Gender			
Female	14 (63.6%)	8 (36.4%)	0.909
Male	18 (62.1%)	11 (37.9%)	
BMI (kg/m^2^)	23.81 ± 0.7	23.67 ± 0.6	0.891
CRP	16.45 ± 2.6	15.46 ± 2.4	0.800
Leukocytes	12.64 ± 1.2	13.63 ± 2.4	0.684
Lactate	1.92 ± 0.2	1.98 ± 1.1	0.871
SvO_2_	70.44 ± 2.3	73.89 ± 2.6	0.344
SOFA	8.00 ± 0.5	7.95 ± 0.8	0.956
APACHE	12.9 ± 1.1	12.68 ± 1.6	0.909
Sepsis Status			
Septic	14 (63.6%)	8 (36.4%)	0.909
Non-Septic	18 (62.1%)	11 (37.9%)	
OS			
Alive	29 (82.9%)	6 (17.1%)	**<0.0001**
Death	3 (18.8%)	13 (81.3%)	

CRP, C Reactive protein; SvO_2_, Mixed venous oxygen saturation; SOFA, Sequential Organ Failure Assessment; APACHE, Acute Physiology and Chronic Health Evaluation; the values are shown as mean ± standard deviation; *p* < 0.05 was considered significant and are denoted in bold; *p*-values were calculated by a student’s *t*-test.

## Data Availability

All data is contained within the article.
